# TKTL1 participated in malignant progression of cervical cancer cells via regulating AKT signal mediated PFKFB3 and thus regulating glycolysis

**DOI:** 10.1186/s12935-021-02383-z

**Published:** 2021-12-18

**Authors:** Yingping Zhu, Yu Qiu, Xueqin Zhang

**Affiliations:** 1 Department of Obstetrics and Gynecology, The First Affiliated Hospital of Zhejiang University of Traditional Chinese Medicine, Hangzhou, 310006 Zhejiang China; 2 Department of Obstetrics and Gynecology, Women and Children’s Hospital, School of Medicine, Xiamen University, NO.10 Zhenhai Road, Siming District, Xiamen, 361000 Fujian China

**Keywords:** TKTL1, Glycolysis, Cervical cancer, Malignant progression, AKT signal, HK2, PFKFB3

## Abstract

**Background:**

Cervical cancer (CC) is the second most common cancer among women with high morbidity and mortality. TKTL1 is a key protein in glucose metabolism in cancer cells and controls the pentose phosphate pathway (PPP). In this paper, we aim to explore whether TKTL1 can participate in the malignant process of CC cells through glucose metabolism.

**Methods:**

The expression and activity of TKTL1 in CC cell lines were detected by RT-qPCR and Western blot. Cell transfection was conducted to interfere the expression of TKTL1 in SiHa cells, with efficiency detected by RT-qPCR and Western blot. Cell proliferation was then measured by CCK-8 kits. Wound Healing and Transwell experiments were performed to respectively detect the levels of cell migration and invasion, and western blot was used to detect the expressions of migration-related proteins. Tunel and Western blot were used to detect the apoptosis and apoptosis-related proteins. Glucose uptake, lactate production, and ATP production were measured by corresponding commercial kits. Next, the expression of p-Akt, AKT, p-MTOR, mTOR, HK2 and PFKFB3 was detected by Western blot. The mechanism was further investigated by interfering the expression of HK2 and PFKFB3 and adding AKT agonist SC79. At the animal level, the tumor bearing mouse model of CC was constructed, and the weight, volume and pathological morphology of the tumor tissue were detected to verify the cell experiment.

**Results:**

TKTL1 expression was increased in CC cells. Interference of TKTL1 expression can inhibit TKTL1 enzyme activity, proliferation, invasion and migration of CC cells, and simultaneously suppress the generation of glycolysis. In addition, the results showed that TKTL1 activated PFKFB3 through AKT rather than HK2 signaling and is involved in glycolysis, cell invasion, migration, and apoptosis of CC cells. In animal level, inhibition of TKTL1 also contributed to decreased tumor volume of CC tumor bearing mice and improved histopathological status.

**Conclusion:**

TKTL1 participated in malignant progression of CC cells via regulating AKT signal-mediated HK2 and PFKFB3 and thus regulating glucose metabolism.

## Introduction

Cervical cancer (CC) is the second most common cancer among women [1]. There are about 529,800 new diagnosed CC cases and 275,100 deaths from CC each year [2]. Moreover, CC remains one of the most common cancers with high prevalence in most less developed countries and one of the leading causes of cancer deaths among women [3].

At present, the pathogenesis of tumor is relatively complex and diversified. Previous study has shown that metabolic reprogramming is an important marker of physiological changes in tumor cells [4]. Among a wide range of procedures in metabolic reprogramming, not only aerobic glycolysis of tumor cells does meet the energy requirements on rapid proliferation of tumor cells, but it also provides a material basis for the synthesis of biomacromolecules [5]. As an important branch of glucose metabolism, pentose phosphate pathway (PPP) plays an important role in biosynthesis and promotes the process and development of tumors [6, 7]. Tranketolase (TKT) is the most efficient enzyme in the non-oxidative branch of the PPP pathway, and it serves as a key enzyme linking PPP to glycolysis pathways in tumor cells [8]. Transketolase-like-1 (TKTL1) is a rate-limiting enzyme in the non-oxidized part of the PPP [9].The TKTL1 gene is located on chromosome Xq28, which is often activated in malignant tumors [10]. Studies have shown that TKTL1 is responsible for about 60–70% of TKT activity in human liver and colon cancer cells [11–13]. These results indicate that TKTL1 plays an important role in the regulation of tumor metabolism. In addition, TKTL1 has been shown to promote carcinogenesis by enhancing aerobic glycolysis [9]. Therefore, TKTL1 may become a new molecular target for tumor therapy.

Current study has shown that TKTL1 expression, which is increased in human endometrial cancer, is related to AKT phosphorylation level and GLUT1 [14]. In addition, the increased expression of TKTL1 has been proved to be a diagnostic marker in low-grade CC [15]. At the same time, study has confirmed that TKTL1 expression is increased in CC and can be used as a diagnostic marker for CC [16]. Since TKTL1 has a cancer-promoting biological activity, this paper aims to explore whether TKTL1 can participate in the malignant process of CC cells through the glucose metabolism pathway.

The oncogene AKT plays an important role in glycolysis during tumorigenesis. The hyperactivation of PTEN/PI3K/AKT/mTOR pathway in bladder cancer plays a central regulatory role in aerobic glycolysis, thereby promoting tumor metabolic switch and tumor cell proliferation [17]. Fdft1-mediated AKT/mTOR/ HIF1α pathway can inhibit aerobic glycolysis and proliferation of colorectal cancer [18]. Study has shown that TKTL1 is significantly correlated with the expression level of phosphorylated AKT in star glioma tissues [19]. In addition, Nico Kohrenhagen et al. showed that TKTL1 and P-Akt play an important role in the occurrence and development of CC, and they speculated that this might be attributed to the effect of TKTL1 and P-Akt on glycolysis [20].Therefore, in our experiment, the relationship between TKTL1 and AKT in CC, its influence on glycolysis in CC and the specific mechanism will be discussed.

## Material and methods

### Cell culture

The human normal cell line End1/E6E7 and CC cell lines SiHa, C33A and HT-3 cells were obtained from the American Type Culture Collection (ATCC). All cells were cultured in DMEM (Gibco, USA) supplemented with 10% FBS (Hyclone, USA) at 37 ℃ with 5% CO_2_.

### Cell transfection

SiHa cells were seeded in 24-well cell culture plates (5 × 10^4^ cells/well) overnight. Cells were then transfected with appropriate concentrated lentivirus TKTL1, HK2 and PFKFB3 shRNA or their control shRNA as per the manufacturer’s instructions, Santa Cruz Biotechnology, Inc. (Santa Cruz, CA). The stably transfected SiHa cells were selected with puromycin (2 μg/ml). RT-qPCR and Western blot were used to detect the interference.

### Tumorigenicity assays in nude mice

The mice were randomly divided into 2 groups (n = 5): Control, shRNA-TKTL1 and shRNA-TKTL1 + SC-79. 2 × 10^6^ transfected SiHa cells (suspended in 0.1 ml of PBS) were injected subcutaneously into the right armpit of the Balb/c nude mice (6-week-old, Female). Mice in shRNA-TKTL1 group were injected with SiHa cells with shRNA-TKTL1 plasmid, with three times a week for six weeks. The weight and the tumor diameter of each mouse were measured every week. Tumor volume (mm3) was calculated as follows: (shortest diameter) 2 × (longest diameter) × 0.5. All mice were subsequently euthanized. The mice were put into the euthanasia box, and carbon dioxide was poured into the box at a rate of 10–30% of the volume of the euthanasia box every minute. 5 min later, it was confirmed that the mice were immobile and did not breathe, and carbon dioxide was closed after pupil dilation. Animal death was confirmed after observation for 2 min. All animal experiments were approved by the Ethics Committee for Laboratory Animals of The First Affiliated Hospital of Zhejiang University of Traditional Chinese Medicine.

### RT-qPCR

RT-qPCR was conducted to quantify the expression of mRNA. Briefly, total RNA from cells was extracted using TRIzol reagent (Invitrogen) according to the protocol as described by the manufacturer. Aliquots of total RNA (1 μg) were reverse-transcribed into cDNA using RevertAid First-Strand cDNA Synthesis Kit (TaKaRa, Tokyo, Japan) according to the instructions of the first-strand cDNA synthesis kit manufacturer. Equal amounts of the reverse transcriptional products were subjected to PCR amplification, using SYBR®Premix Ex TaqII RT-PCR Kit (TaKaRa). The mRNA levels of target genes were normalized to the GAPDH mRNA levels. The primers used in this study were synthesized by Operon, and the sequences were as follows (5′- 3′): TKTL1, sense TCTCCGAACTGCAAGTGCTA and antisense CTGCAAACTTTTGGAGAGCA; HK2, sense TCTCCGAACTGCAAGTGCTA and antisense CTGCAAACTTTTGGAGAGCA; PFKFB3, sense TCTCCGAACTGCAAGTGCTA and antisense CTGCAAACTTTTGGAGAGCA; and for GAPDH, sense TCGCTGCGCTGGTCGTC and antisense GGCCTCGTCACCCACATAGGA. Relative quantification of genes was analyzed in accordance with the 2 − ΔΔCt method (ΔΔCt = ΔCt [treated] − ΔCt [control]). β-actin was used as endogenous control.

### Western blot

Cells were lysed in RIPA buffer. Protein concentrations were determined using the Bradford Assay (Bio‐Rad). 30 μg protein lysates were then subjected to 12% Tris‐glycine SDS PAGE, and then transferred onto PVDF membranes (Invitrogen) according to the manufacturer’s instructions. The membranes were blocked in 5% skimmed milk powder for 1 h, followed by incubation with primary antibodies at 4 °C overnight. On the following day, the membranes were incubated with HRP‐conjugated secondary antibodies (Cell Signaling) at room temperature for 1 h. Protein expression was visualized by ECL chemiluminescence (Promega) and quantified using Image J software (National Cancer Institute).

### Measurement of TKTL1 activity

SiHa cells with different treatments were sonicated and centrifuged, and the supernatant was collected and filtered to remove some endogenous metabolites. TKTL1 activity was determined using enzyme-linked method and expressed as the ratio of productions per minutes (ng) to total protein (mg). Each experiment was repeated at least thrice.

### CCK-8

The tumor cell viability was assessed by CCK-8 assay. SiHa cells (1 × 10^3^) were seeded into 96-well plates and transfected for 48 h. CCK-8 reagent was added into wells for 3 h using Cell Counting Kit-8 (CCK-8; Beyotime Institute of Biotechnology, Shanghai, China) according to the manufacturer's instructions. Cells viability was analyzed by a microplate reader (Bio-Rad Laboratories, Inc., Hercules, CA, USA) at 450 nm.

### Wound healing

SiHa cells were grown in a 6-well plates until 100% confluence was achieved. Cells were incubated accordingly. A wound was made using 20-μL pipette tip through the cell monolayer. Serial images were obtained at time 0 and 24 h under light microscopy. Wound-healing was evaluated by measuring the total surface area of the image covered by the cells.

### Transwell

SiHa cells were treated with serum-free medium for 12 h, and then were resuspended (5 × 10^5^ cells/well) in DMEM medium containing 1.0% FBS. The cell suspension (20 μL) was added to the upper chamber of a 24-well plate Transwell chamber (Corning, Inc.), with the lower chamber containing 500 μL of 10% FBS DMEM medium. Then, the cells were fixed with 4% paraformaldehyde. The cells that had not migrated on the membrane were removed with a cotton swab, and the cells on the membrane were stained with 1% crystal violet for 5 min, and ten fields under the microscope were randomly selected for counting (magnification × 200).

### TUNEL

Cell apoptosis was detected using a TUNEL assay kit (Roche Diagnostics GmbH, Germany) according to the manufacturer's instructions. Briefly, The cells are treated accordingly and were then digested with 20 μg/mL proteinase K for 5 min, rinsed with PBS and incubated with TUNEL reagents containing terminal deoxynucleotidyl transferase (TdT) and fluorescent isothiocyanate dUTP for 2 h at 37 °C. Finally, the samples were stained with DAPI for 30 min to evaluate the cell nucleus. The apoptotic cells were recognised with dual TUNEL and DAPI staining under a fluorescence and UV light microscope.

### Metabolism measurements

The extracellular acidification rate (ECAR) was determined in real-time using the Seahorse XFe24 according to the manufacturer’s instructions (North Billerica, USA). Glucose uptake, lactate production, and ATP production were measured using commercial assay kits (Jiancheng, China) according to the manufacturer’s instruction and values were normalized to the concentration of protein.

### Hematoxylin and eosin (H&E) staining

Tumour tissues of mice were isolated and fixed in 10% formalin at room temperature for24h. The tissues were dehydrated, paraffin embedded and then cut into 5‐μm‐thick sections for hematoxylin and eosin (H&E) staining.

### Statistical analysis

Experimental results are presented as mean ± SD from at least three separate experiments. Differences between groups were analyzed using Student’s *t*-test, one-way ANOVA with Tukey’s post hoc test. Statistical analyses were performed using SPSS software (version 11.0). *P-*values < 0.05 were considered statistically significant.

## Results

### TKTL1 interference inhibited the malignant progression of of CC cells

**Fig. 1:**
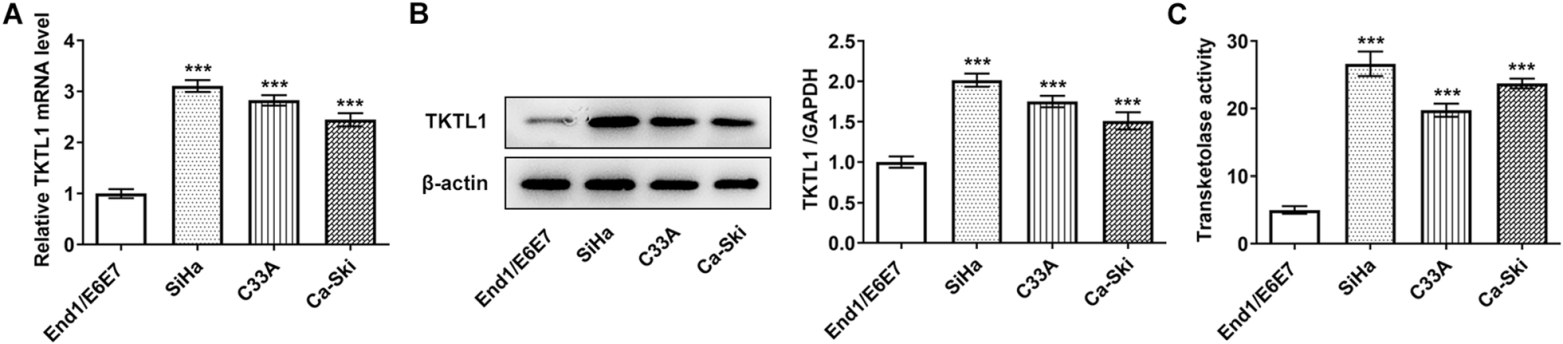
The expression of TKTL1 in the CC cells. **A** RT-qPCR detected the expression of TKTL1 in the CC cells. **B** Western blot detected the expression of TKTL1 in the CC cells. **C** Enzyme-linked method was used to detect the transketolase activity. ***p < 0.001 vs End1/E6E7

**Fig. 2:**
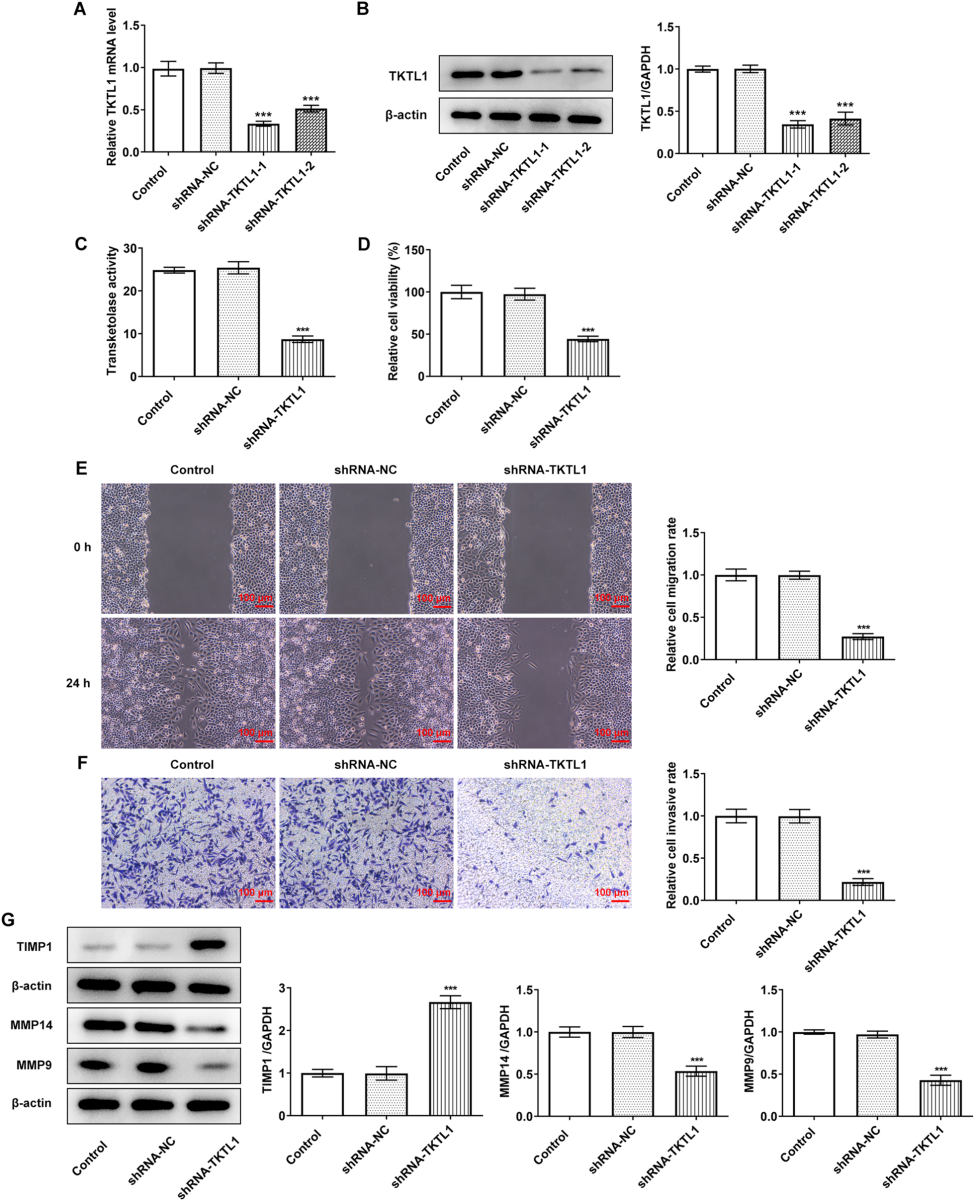
TKTL1 interference inhibited the proliferation, invasion and migration of CC cells. **A** RT-qPCR detected the expression of TKTL1 in the CC cells after cell transfection of shRNA-TKTL1. **B** Western blot detected the expression of TKTL1 in the CC cells after cell transfection of shRNA-TKTL1. **C** Enzyme-linked method was used to detect the transketolase activity after cell transfection of shRNA-TKTL1. **D** CCK-8 detected the cell viability after cell transfection of shRNA-TKTL1. **E** Wound healing detected the migration of cells after transfection of shRNA-TKTL1. **F** Transwell detected the invasion of cells after transfection of shRNA-TKTL1. **G** Western blot detected the expression of TIMP1, MMP14 and MMP9. ***p < 0.05 vs shRNA-NC

The results of RT-qPCR and Western blot showed that TKTL1 expression in CC cell lines was abnormally increased (Fig. 1A and B). The activity of TKTL1 in the cells was then determined by enzymelinked assay, which showed a significant increase in TKTL1 activity in CC cells compared with End1/E6E7 cells (Fig. 1C). We selected SiHa cells with the highest TKTL1 expression level and enzyme activity for the next experimental study. The transfection efficiency was detected by RT-qPCR, Western blot and enzymelinked assay. The results showed that the expression of TKTL1 in cells of shRNA-TKTL1-1 and shRNA-TKTL1-2 groups was significantly decreased compared with that of shRNA-NC group, and the enzyme activity of TKTL1 was also inhibited (Fig. 2A, B and C). Since the inhibitory effect of shRNA-TKTL1-1 was more obvious, we selected shRNA-TKTL1-1 for the next experiment. Cell proliferation was measured by CCK-8, and we found that cell proliferation was significantly decreased in the shRNA-TKTL1 group compared with the shRNA-NC group (Fig. 2D). Wound Healing and Transwell results showed that compared with the shRNA-NC group, the cell migration and invasion of the shRNA-TKTL1 group were significantly decreased (Fig. 2E and F), accompanied by decreases in proteins MMP14 and MMP9, and an increase in TIMP1 (Fig. 2G). Our results showed that TKTL1 interference inhibited the proliferation, invasion and migration of CC cells.

**Fig. 3:**
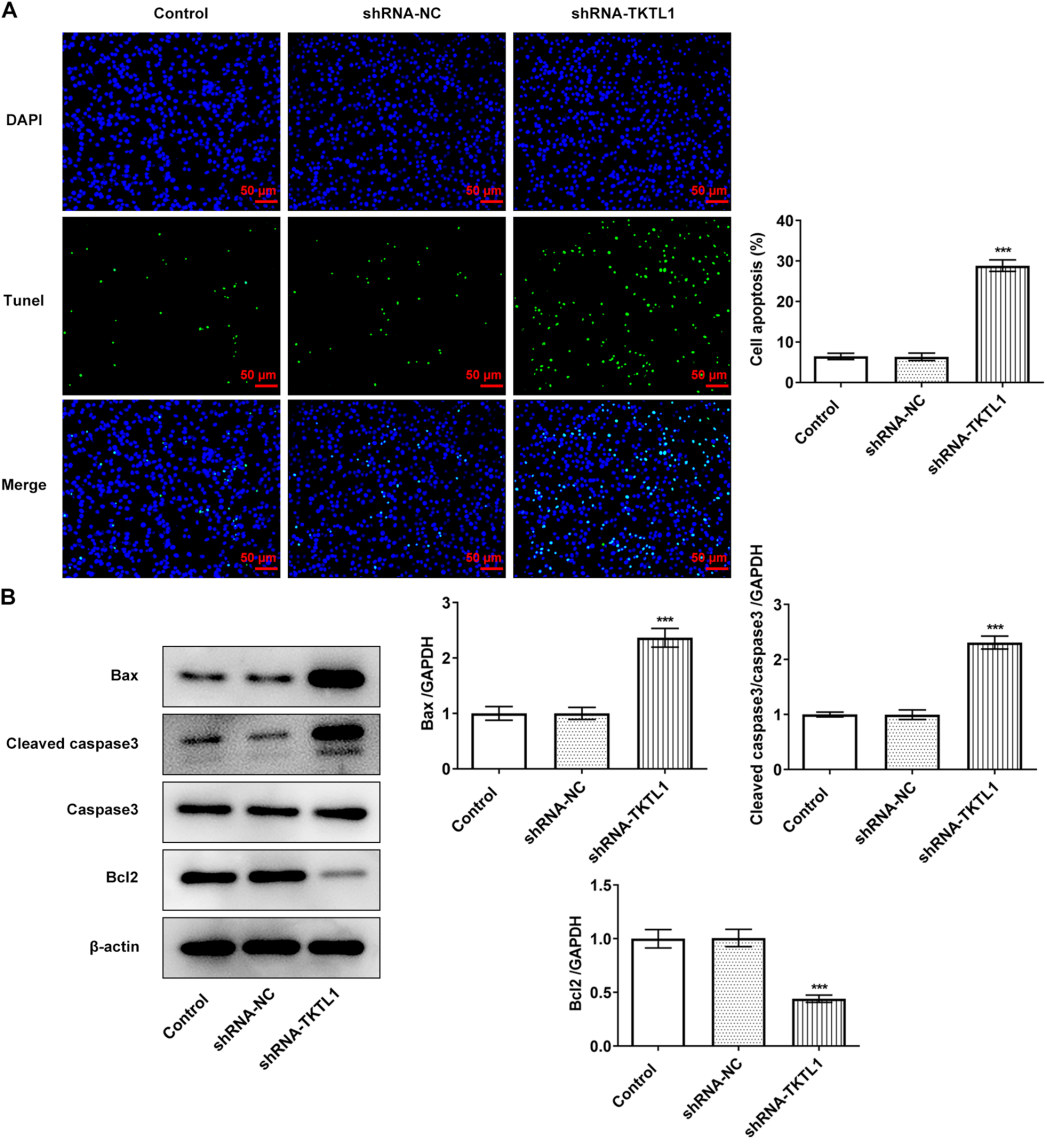
TKTL1 interference promoted apoptosis of CC cells. **A** Tunel assay detected the apoptosis of cells after transfection of shRNA-TKTL1. **B** Western blot detected the expression of apoptosis-related proteins. ***p < 0.05 vs shRNA-NC

Regarding the cell apoptosis level, Tunel and Western blot results showed that compared with the shRNA-NC group, apoptosis of cells in the shRNA-TKTL1 group was significantly increased (Fig. 3A), accompanied by significant increases in Bax and Cleaved caspase3 and a significant decrease in Bcl2 (Fig. 3B).

### TKTL1 interference inhibited the production of glycolysis of CC cells

**Fig. 4:**
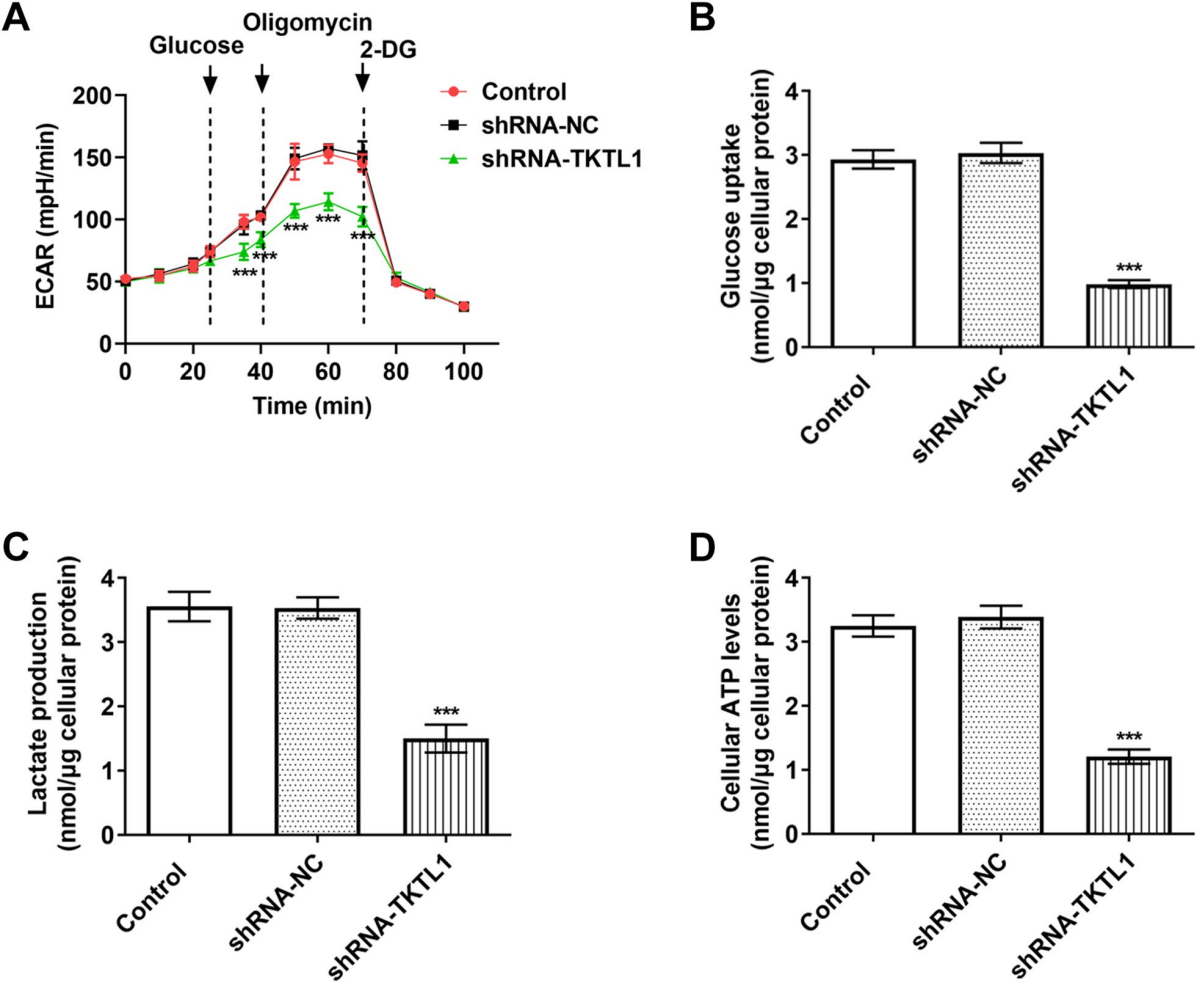
TKTL1 interference inhibited the production of glycolysis in CC cells. **A** The effect of TKTL1 interference on the extra cellular acidification rate (ECAR) in SiHa cells. The effect of TKTL1 interference on glucose uptake (**B**), lactate production (**C**), and ATP generation (**D**) in SiHa cells. ***p < 0.05 vs shRNA-NC

In order to study the role of TKTL1 in the aerobic glycolysis of CC, we detected the extra cellular acidification rate (ECAR). The results showed that compared with the shRNA-NC group, the shRNA-TKTL1 group showed a significant decrease in glycolysis (Fig. 4A).In addition, TKTL1 interference attenuated glucose uptake, lactic acid production and ATP production levels in siHa cells compared with the control group (Fig. 4B, C and D).

### TKTL1 interference inhibited the expression of AKT, HK2 and PFKFB3 of CC cells

**Fig. 5:**
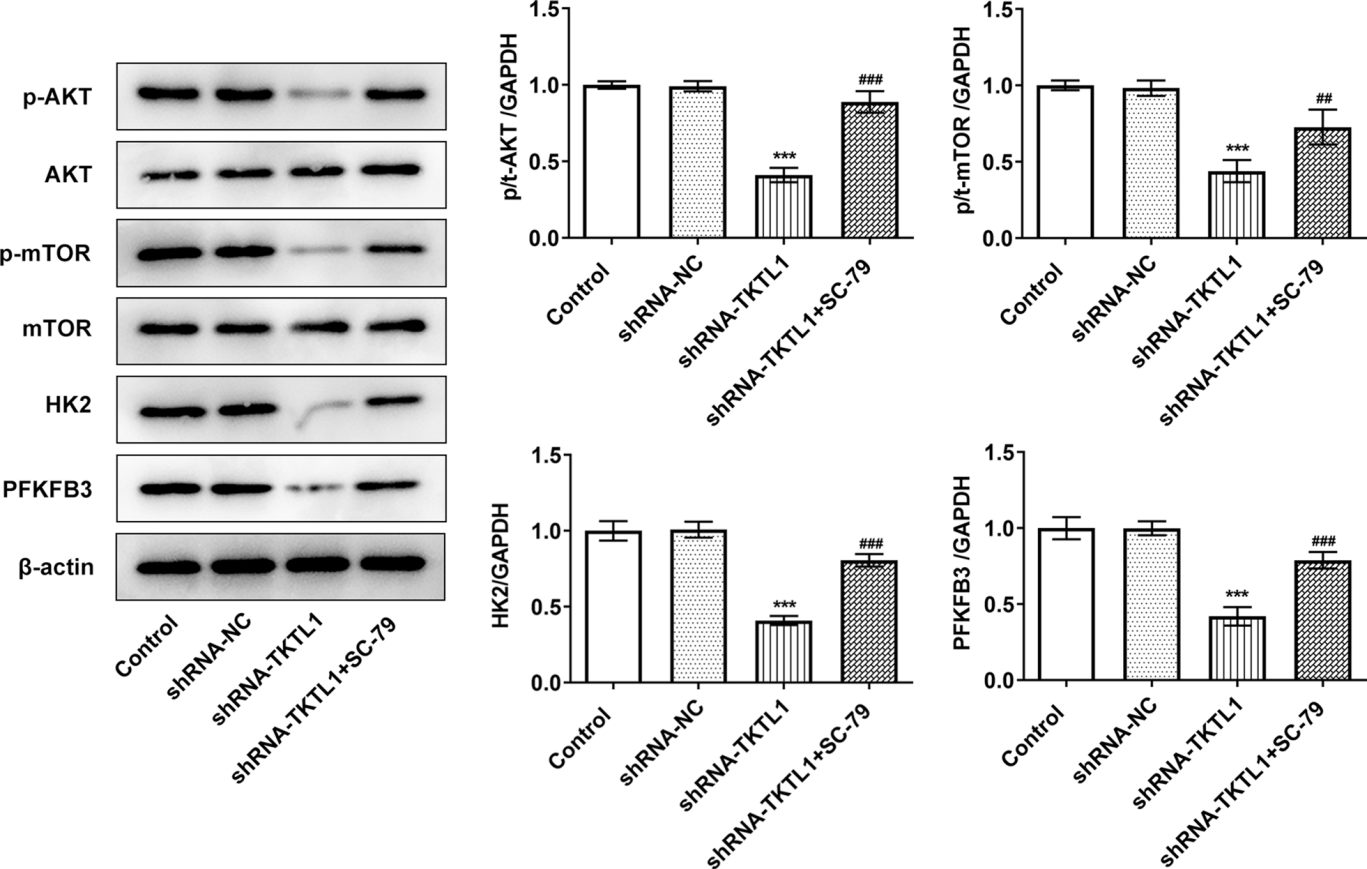
TKTL1 interference inhibited the expression of AKT signaling pathway proteins, HK2 and PFKFB3 in CC cells. Western blot detected the expression of AKT signaling pathway related proteins, HK2 and PFKFB3. ***p < 0.05 vs shRNA-NC, ##p < 0.01, ###p < 0.001 vs shRNA-TKTL1

During the experiment, we found that the expression of AKT signaling pathway was abnormal. Compared with the shRNA-NC group, the expression of p-Akt and p-mTOR in shRNA-TKTL1 group was significantly decreased, and the expression of HK and PFKFB3 was significantly decreased. The expression of p-Akt, p-mTOR, HK and PFKFB3 was significantly increased after the addition of SC-79, an AKT signaling pathway inhibitor, compared with the shRNA-TKTL1 group (Fig. 5). This suggested that TKTL1 may regulate the expression of key enzymes HK and PFKFB3 in glycolysis through AKT signaling pathway.

### TKTL1 regulated glycolysis of CC cells via AKT/PFKFB3 pathway

**Fig. 6:**
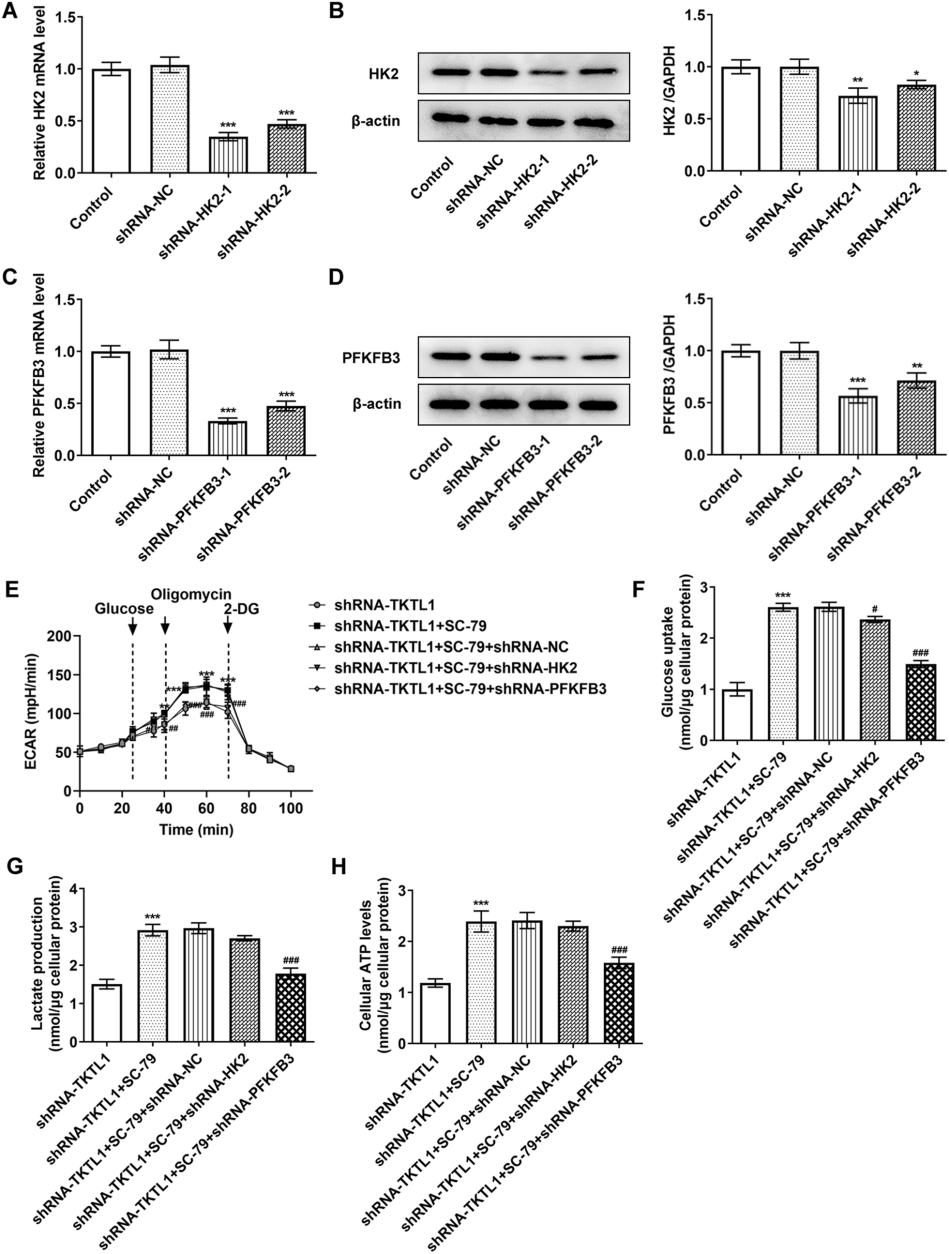
TKTL1 was involved in glycolysis of CC cells by regulating AKT and thus activating PFKFB3 rather than HK2. **A** RT-qPCR detected the expression of HK-2 in the CC cells after transfection of shRNA-HK-2. **B** Western blot detected the expression of HK-2 in the CC cells after transfection of shRNA-HK-2. **C** RT-qPCR detected the expression of PFKFB3 in the CC cells after transfection of shRNA- PFKFB3. **D** Western blot detected the expression of PFKFB3 in the CC cells after transfection of shRNA-PFKFB3. *p < 0.05, **p < 0.01, ***p < 0.001 vs shRNA-NC. **E** The effect on the ECAR in SiHa cells after cell transfection. The effect on glucose uptake (**F**), lactate production (**G**), and ATP generation (**H**) in SiHa cells after cell transfection. ***p < 0.001 vs shRNA-TKTL1; #p < 0.05, ##p < 0.01, ###p < 0.001 vs shRNA-TKTL1 + SC-79 + shRNA-NC

To further investigate the mechanism, we inhibited the expression of HK2 and PFKFB3 in cells by cell transfection assay. Compared with the shRNA-NC group, the expression of HK2 in shRNA-HK2-1 and shRNA-HK2-2 decreased significantly, and the shRNA-HK2-1 group showed the most significant decrease, so shRNA-HK2-1 was selected for the next experiment (Fig. 6A and B). Similarly, shRNA-PFKFB3-1 was selected for the following experiment (Fig. 6C and D). We grouped the cells into shRNA-TKTL1, shRNA-TKTL1 + SC-79, shRNA-TKTL1 + SC-79 + shRNA-NC, shRNA-TKTL1 + SC-79 + shRNA-HK2 and shRNA-TKTL1 + SC-79 + shRNA-PFKFB3. We detected the ECAR, and the results showed that compared with the shRNA-TKTL1 group, the glycolysis ability of the shRNA-TKTL1 + SC-79 group was significantly increased. Compared with shRRNA-TKTL1 + SC-79 + shRNA-NC, the glycolysis ability, glucose uptake, lactic acid production and ATP production levels of shRNA-TKTL1 + SC-79 + shRNA-HK2 did not change significantly. The glycolysis capacity of the shRNA-TKTL1 + SC-79 + shRNA-PFKFB3 group was significantly decreased (Fig. 6E), accompanied by decreased glucose uptake, lactic acid production and ATP production levels (Fig. 6F, G and H). These results indicated that TKTL1 activated PFKFB3 rather than HK2 through AKT signaling in the production of glycolysis of CC cells.

### TKTL1 regulated the malignant progression of CC cells via AKT/PFKFB3 pathway

**Fig. 7:**
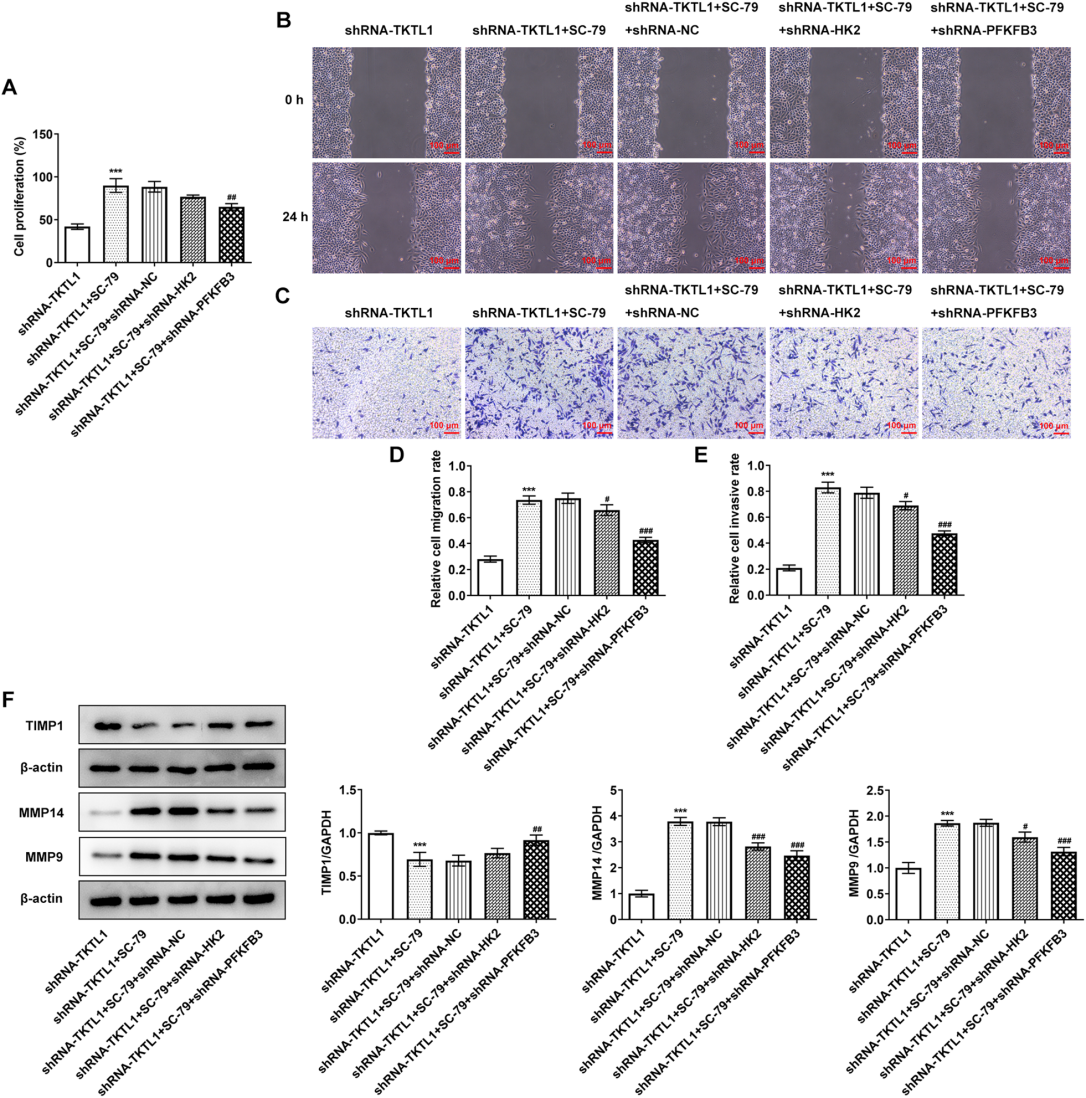
TKTL1 was involved in proliferation, invasion and migration of CC cells by regulating AKT and thus activating PFKFB3 rather than HK2. **A** CCK-8 detected the cell viability after cell transfection. **B** Wound healing detected the migration of cells after transfection. **C** Transwell detected the invasion of cells after transfection. **D** Statistical diagram of cell migration. **E** Statistical diagram of cell invasion. **F** Western blot detected the expression of TIMP1, MMP14 and MMP9. ***p < 0.001 vs shRNA-TKTL1; #p < 0.05, ##p < 0.01, ###p < 0.001 vs shRNA-TKTL1 + SC-79 + shRNA-NC

**Fig. 8:**
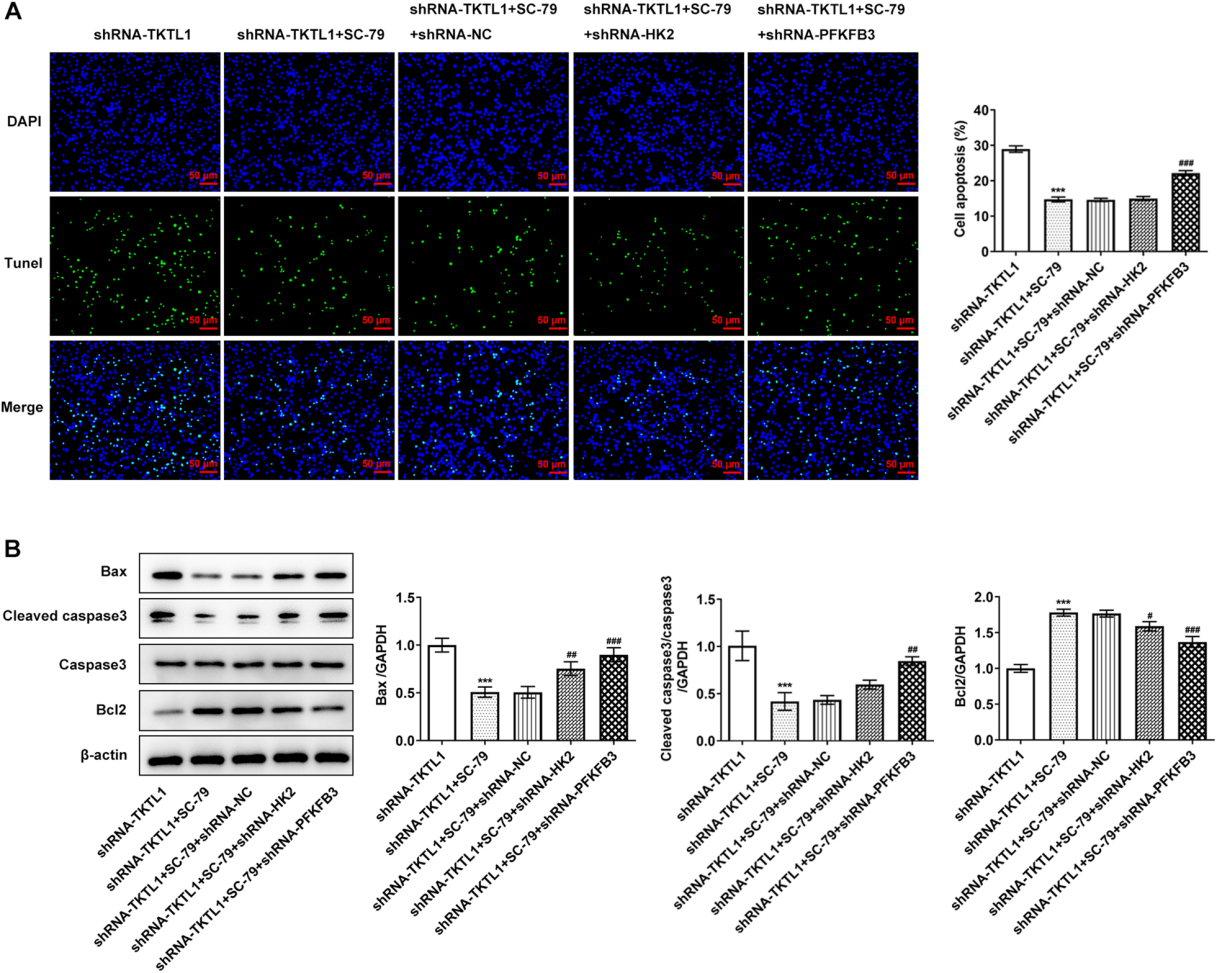
TKTL1 was involved in apoptosis of CC cells by regulating AKT and thus activating PFKFB3 rather than HK2. **A** Tunel assay detected the apoptosis of cells after transfection. **B** Western blot detected the expression of apoptosis-related proteins. ***p < 0.001 vs shRNA-TKTL1; ##p < 0.01, ###p < 0.001 vs shRNA-TKTL1 + SC-79 + shRNA-NC

The malignant progression of CC was then detected. The results showed that compared with shRNA-TKTL1 + SC-79 + shRNA-NC, the proliferation, invasion and migration of shRNA-TKTL1 + SC-79 + shRNA-HK2 cells were not significantly changed. The cell proliferation (Fig. 7A), invasion and migration of shRNA-TKTL1 + SC-79 + shRNA-PFKFB3 group were significantly decreased (Fig. 7B–F). Tunel and Western blot results showed that compared with shRNA-TKTL1 + SC-79 + shRNA-NC, shRNA-TKTL1 + SC-79 + shRNA-HK2 cells had no significant change in apoptosis. shRNA-TKTL1 + SC-79 + shRNA-PFKFB3 group showed a significantly increased apoptosis (Fig. 8A), accompanied by increased Bax, cleaved caspase3 protein, decreased Bcl2 protein (Fig. 8B).

### TKTL1 was involved in the growth of CC

**Fig. 9:**
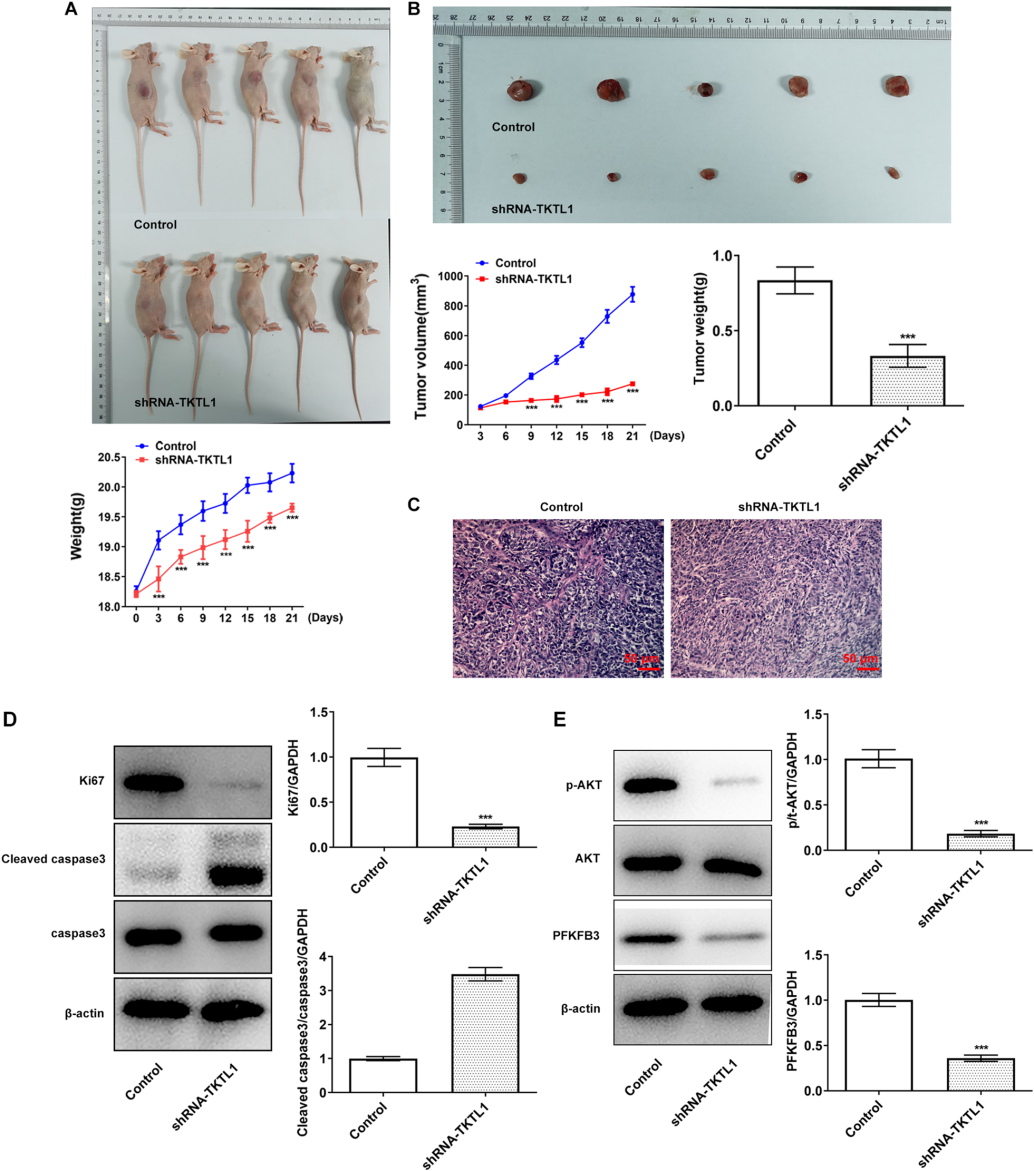
TKTL1 was involved in the growth of CC. **A** Statistical analysis of mouse body weight. **B** Volume and weight of tumor tissue. **C** HE staining was used to detect the pathological morphology of the tumor. **D** Western blot detected the expression of Ki67, caspase3 and cleaved caspase3. **E** Western blot detected the expression of p-AKT, AKT and PFKFB3. ***p < 0.001 vs control

We inhibited the expression of TKTL1 in tumor bearing mice of CC. After treatment, the mice were photographed and tumor tissues were taken. We found that mice in the shRNA-TKTL1 group had a significant weight loss compared with the control group (Fig. 9A). The tumor tissue was taken out and photographed, with volume and weight calculated. We found significant decreases in tumor volume and weight of mice in the shRNA-TKTL1 group compared with the control group (Fig. 9B). HE staining results showed that the tissues of the control group were normal, and the tumor histopathology of the shRNA-TKTL1 group was changed (Fig. 9C). The expression of proliferation related protein Ki67 and apoptosis related protein Cleaved caspase3 and Caspase3 were detected by Western blot, and the results showed that the expression of Ki67 was decreased in the SHRNA-TKTL1 group compared with the control group. And cleaved caspase3 expression was significantly increased (Fig. 9D). The expression of p-AKT, AKT and PFKFB3 in tumor tissues was also detected at the animal level, and we found that the expression of p-AKT and PFKFB3 was significantly decreased in the shRNA-TKTL1 group compared with the control group (Fig. 9E), which was consistent with the results of cell experiments.

## Discussion

The high proliferative activity and invasiveness of CC directly lead to poor prognosis. Therefore, it is of obvious clinical significance to actively study the mechanism of CC proliferation, invasion and metastasis and seek corresponding therapeutic targets for improving the overall curative effect of CC. Study has shown that the maintenance of biological characteristics of tumor cells requires energy, and glycolysis is the main energy metabolism pathway of tumor cells [21]. A previous study showed that the increase in TKT activity of tumor cells, mainly depending on the high expression of TKTL1, provides sufficient raw materials for nucleic acid synthesis for the large proliferation of tumor cells [22].A previous study indicated abnormally elevated expression of TKTL1 in prostate cancer, colorectal cancer and ovarian cancer, indicative of its potential involvement in cancer development [23–25].In our experiment, it was found that the expression of TKTL1 in CC cell lines was also significantly increased, which was consistent with the results of other studies.In recent years, a large number of clinical studies have shown that the high expression of TKTL1 was often closely related to tumor invasion, metastasis and poor prognosis, so people have begun paying attention to the relationship between TKTL1 and tumor malignant phenotype and trying to reveal its internal mechanism.Li et al. [26] showed that TKTL1 promoted cell proliferation and metastasis in esophageal squamous cell carcinoma. TKTL1 was highly expressed in melanoma cells and promoted the invasion of melanoma cells [27].In our experiment, it was found that TKTL1 interference inhibited the proliferation, invasion and migration of CC cells and promoted cell apoptosis. The results indicated that the high expression of TKTL1 was closely related to the invasion and metastasis of CC cells.

The proliferation of tumor cells is closely related to the abnormal glucose metabolism [28]. In the metabolic process of many malignant tumors, even under sufficient oxygen supply, they prefer the glycolysis pathway, which consumes more glucose [29].TKTL1 was a key enzyme in the non-oxidative branch of PPP, and its overexpression promoted the formation of nucleic acid, ATP and lactic acid, thus providing a large amount of raw materials for tumor growth [9]. Our data also showed that the inhibition of TKTL1 expression reduced the production of glycolysis in CC cells, together with the blocked malignant process of CC cells.

Study has shown that TKTL1 was significantly correlated with the expression level of p-AKT in star glioma tissues [30]. TKTL1 and p-AKT play important roles in the occurrence and development of CC [20]. In addition, the oncogene AKT played an important role in glycolysis during tumorigenesis [18]. In our experiments, it was found that the expression of p-Akt and p-mTOR in cells decreased significantly after the inhibition of TKTL1 expression. Further addition of AKT agonist SC-79 resulted in increased glycolysis, increased cell proliferation, invasion and migration, and decreased apoptosis. These results indicated that TKTL1 can regulate the glycolysis level of CC cells by inhibiting the phosphorylation of AKT pathway, thus regulating the malignant process of CC cells.

Hexokinase (HK) is the first rate-limiting enzyme that controls glycolysis, whose subtype HK-2 is highly expressed in various tumors [31]. Since both C-terminal and N-terminal of HK-2 retain catalytic activity, the process of glycolysis is greatly accelerated by HK-2 [32]. After the knockout of HK-2 in gastric cancer cells, the proliferation, migration and invasion of the tumors were significantly reduced [33]. This indicated that HK-2 played an important role in tumor glycolysis and malignant process. AKT regulated glucose metabolism, and promoted glycolysis by accelerating the transport of GLUT1 to the cell surface and by activating phosphofructokinase (PFK) and HK [6]. In our experiments, it was found that the expression of HK-2 in cells decreased after the inhibition of TKTL1 expression while increased after further addition of SC-79. However, after depletion of HK-2 in the cells, there was no significant change in the glycolysis level and malignant progression of CC cells. These results indicated that TKTL1 could affect the glycolysis and malignant process of tumor cells by regulating AKT rather than activating the expression of HK-2. The change in HK-2 expression was attributed to the affected overall glycolysis but not the AKT regulation.

Glycolysis regulatory enzymes such as phosphofructokinase 2(PFK2) increase glycolysis flux by activating PFK1, whose subtype PFKFB3, which is expressed in many cancers, is a key rate-limiting enzyme that controls glycolysis [34]. Study has shown that inhibition of PFKFB3 can inhibit the proliferation, migration and invasion of breast cancer cells (MDA-MB-231 and MDA-MB-468), and induce cell cycle G1 and S phase in vitro arrest [35]. Moreover, AKT can control the expression of PFKFB3 and participate in glycolysis [7].Our experiment showed that the expression of PFKFB3 decreased after inhibition of TKTL1 expression. After further activating AKT expression, PFKFB3 expression was reversed. Therefore, we further inhibited the expression of PFKFB3 in cells under the condition of inhibiting the expression of TKTL1 and activating the expression of AKT. We found that after further inhibiting the expression of PFKFB3, the glycemic ability of cells was significantly decreased, cell proliferation, invasion and migration were inhibited, and cell apoptosis was increased.

Overall, our results indicated that TKTL1 was involved in proliferation, invasion, migration and apoptosis of CC cells by regulating AKT and thus activating PFKFB3 rather than HK2. Our experimental results provided a solid theoretical basis for targeted therapy of CC.

## Data Availability

The analyzed data sets generated during the present study are available from the corresponding author on reasonable request.
